# Targeted Molecular Imaging of Pancreatic Cancer with a Miniature Endoscope

**DOI:** 10.3390/app7121241

**Published:** 2017-11-30

**Authors:** Xianjin Dai, Weiping Qian, Hao Yang, Lily Yang, Huabei Jiang

**Affiliations:** 1 J. Crayton Pruitt Family Department of Biomedical Engineering, University of Florida, Gainesville, FL 32611, USA; 2 Departments of Surgery, Emory University School of Medicine, Atlanta, GA 30322, USA; 3 Department of Medical Engineering, University of South Florida, Tampa, FL 33620, USA; 4 Departments of Radiology and Imaging Sciences, Emory University School of Medicine, Atlanta, GA 30322, USA

**Keywords:** photoacoustic imaging, fluorescence imaging, multifunctional nanoparticles, pancreatic cancer, molecular imaging, multimodal endoscopy

## Abstract

It is highly desirable to develop novel approaches to improve patient survival rate of pancreatic cancer through early detection. Here, we present such an approach based on photoacoustic and fluorescence molecular imaging of pancreatic tumor using a miniature multimodal endoscope in combination with targeted multifunctional iron oxide nanoparticles (IONPs). A novel fan-shaped scanning mechanism was developed to minimize the invasiveness for endoscopic imaging of pancreatic tumors. The results show that the enhancements in photoacoustic and fluorescence signals using amino-terminal fragment (ATF) targeted IONPs were ~four to six times higher compared to that using non-targeted IONPs. Our study indicates the potential of the combination of the multimodal photoacoustic-fluorescence endoscopy and targeted multifunctional nanoparticles as an efficient tool to provide improved specificity and sensitivity for pancreatic cancer detection.

## 1. Introduction

Pancreatic cancer is the fourth leading cause of cancer-related death in the United States (US). It is estimated that 53,670 new cases will be diagnosed and 43,090 pancreatic cancer patients will die in the US in 2017 [1]. The Pancreatic Cancer Action Network predicts that pancreatic malignancies will become the second leading cause of cancer-related death by 2020 [2]. In pancreatic cancer, the 5-year survival rate is approximately 7%, which is attributed to primary factors including inefficient early diagnostic tools and ineffective treatment methods. Nevertheless, the overall 5-year survival rate is significantly improved to 26% for patients diagnosed in the early stages without metastatic lesions [3]. Therefore, to develop reliable methods that can improve early diagnosis is highly desired.

Conventional diagnostic imaging modalities including X-ray radiography, computed tomography (CT), fluoroscopy, ultrasonography, and magnetic resonance imaging (MRI) have been used for diagnosis and treatment planning for pancreatic cancer medically. However, these conventional imaging modalities provide only structural or anatomical changes, which often happen several years after detrimental molecular changes, especially for pancreas-related diseases. The specificity of these conventional imaging techniques for the early diagnosis of pancreatic cancer is quite low [4].

It is believed that molecular imaging is able to offer highly sensitive and specific detection of tumors through sensing molecular changes. For pancreatic cancer, current existing molecular imaging techniques include positron emission tomography (PET) [5–8], single-photon emission computed tomography (SPECT) [9,10], MRI with contrast agent enhancement (such as magnetic nanoparticles) [11–15], optical/fluorescence imaging [14,16,17], and photoacoustic imaging (PAI) [18–20]. PET and SPECT involve ionization radiation with the long half-life of radiotracers which limits the temporal resolution. In addition, they both have relatively low spatial resolution in localizing the tumors. The relatively slow data acquisition time of MRI often generates motion artifact issue and a reduced signal-to-noise ratio [21]. In optical imaging, fluorescent dyes, quantum dots, or nanoparticles conjugated to targeted antibodies or peptides are commonly used as contrast agents. Optical molecular imaging has a relatively high spatiotemporal resolution without ionization radiation. However, the limited tissue penetration capability of light prevents the use of optical techniques for noninvasive imaging of deeply located organs like the pancreas.

PAI is an emerging biomedical imaging technique that combines the optical contrast with an increased ratio of imaging depth to spatial resolution capable of providing anatomical, functional, molecular properties of biological tissue with a high resolution [22–32]. In PAI, images are formed through detecting pulsed laser-induced wideband acoustic waves. The image contrast in PAI originates from light absorption in tissue. Similar to optical imaging, PAI also suffers from the penetration limitation, and cannot be used to noninvasively image deeply located organs such as the pancreas.

Multifunctional iron oxide nanoparticles (IONPs) have been developed for targeted molecular imaging and drug delivery with their capabilities of tumor targeting, imaging, and delivery of therapeutic agents. IONPs have been widely used to enhance the image contrast for magnetic resonance imaging (MRI) and fluorescence imaging in animals [14,33,34].

The goal of this study is to demonstrate a multimodal endoscopic approach combined with targeted multifunctional IONPs for the molecular imaging of pancreatic tumors, where the pancreas can be directly accessed with minimal invasiveness using a 1 mm-diameter miniature endoscope coupled with a novel fan-shaped two-dimensional (2D) scanning mechanism.

## 2. Materials and Methods

### 2.1. Animal Model and Multifunctional Nanoparticles

In this study, a mouse pancreatic cancer cell line panc02-derived tumor model was used. The pancreatic tumor model was established by injecting pancreatic cancer MIA PaCa-2 cells into the pancreas of 8-week-old female nude mice using a surgical procedure approved by the Institute of Animal Use Committee (IACUC) of Emory University. Strict animal care procedures approved by the Emory University IACUC and based on guidelines from the National Institutes of Health (NIH) guide for the Care and Use of Laboratory Animals were followed. Orthotopically xenografted pancreatic tumors reached several millimeters in diameter and were ready for experiments in about 3 to 4 weeks. Six mice separated into two groups (three in each group) were used in this study to prove the concept of our miniaturized endoscopic imaging system for pancreatic cancer. For systemic delivery, 100 picomoles (pmol) with or without targeting IONPs, with or without polyethylene glycol (PEG) coating IONPs, were injected into the tail vein of the mice once per week for a total of two to four injections. In each group, each mouse received the injection of one of the three types of multifunctional nanoparticles: (1) near-infrared (NIR) 830-maleimide fluorescence dye conjugated to the 10-nm core size IONPs without targeting ligands, NIR830-IONP; (2) NIR830-IONP plus conjugation of amino-terminal fragment (ATF) targeting, NIR830-ATF-IONP; (3) NIR830-ATF-IONP plus polyethylene glycol (PEG) coating, NIR830-ATF-PEG-IONP. Thus, for each type of nanoparticles, two mice were used for the experiments. PEG was used to stabilize the nanoparticles and to modify surface properties to reduce the non-specific uptake of nanoparticles by macrophages in the reticuloendothelial system to improve the targeted delivery of the nanoparticles [34].

### 2.2. Miniaturized Endoscopic Imaging System and Scanning Mechanism

**Figure 1:**
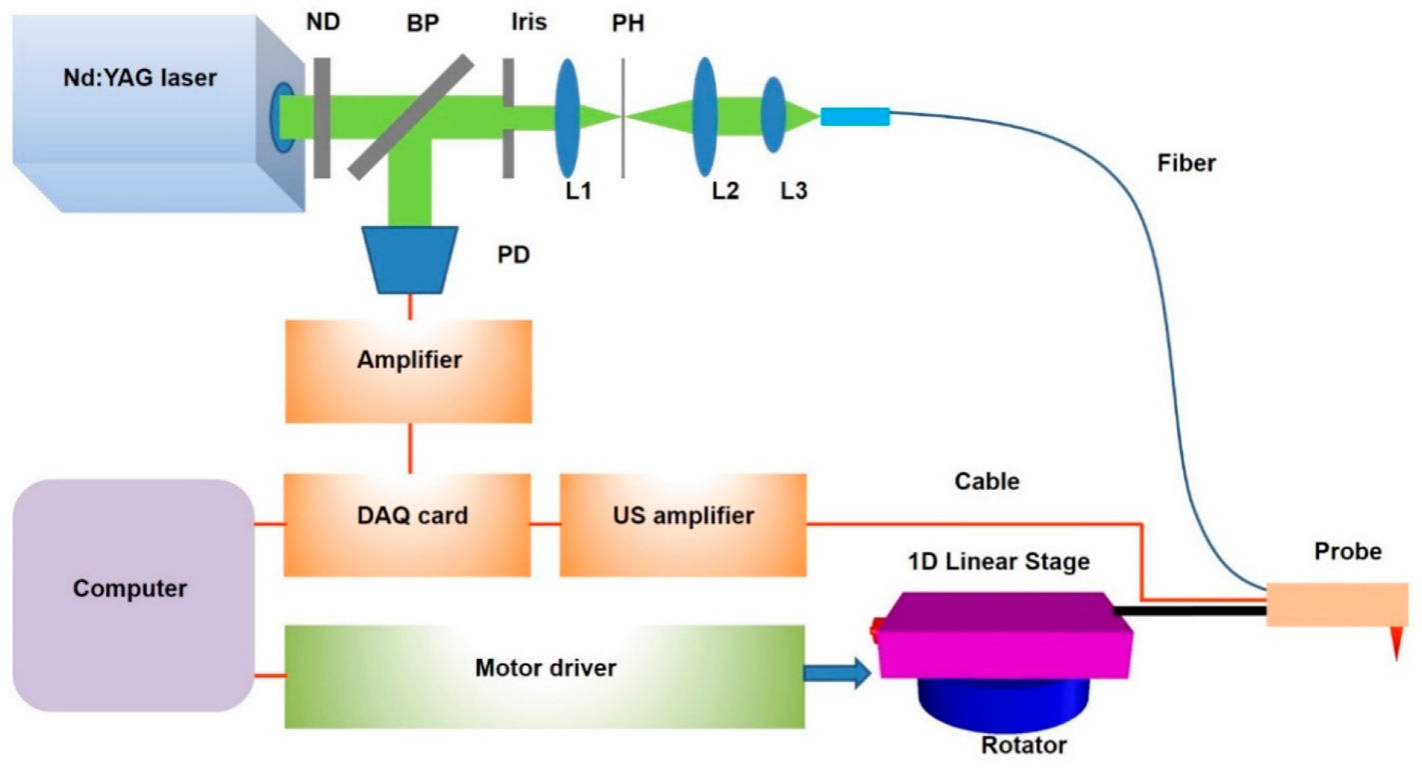
Endoscopic imaging system. ND, neutral density filter; L1, L2, L3, lenses; PH, pinhole; BP, beam splitter; PD, photodiode; DAQ, data acquisition; US, ultrasound.

The multimodal endoscopic probe with a size of 1 mm in diameter described in detail before [35] was used to directly reach the surface of the pancreas through a hole in the abdomen of mouse. Here, photoacoustic imaging was performed first, followed by fluorescence imaging. In brief, a double-clad fiber was integrated into the probe to deliver a light beam, which was then focused by a gradient-index (GRIN) lens with a diameter of 0.25 mm and a working distance of 5 mm. A custom-made unfocused ultrasound transducer with a center frequency of 40 MHz and dimensions of 0.6 mm *×* 0.5 mm *×* 0.2 mm was used to detect the photoacoustic signals. The imaging system is schematically shown in Figure 1. The probe was mounted to the stage consisting of a one-dimensional (1D) linear stage and a rotator. A nanosecond-pulsed Nd:YAG pumped Optical Parametric Oscillator (OPO) laser with a repetition frequency of 20 Hz was used as the light source. The light beam, attenuated by a neutral density (ND) filter, was then split into two parts. One part reached the photodiode (PD) module to monitor light intensity in real time for calibration. The other part, shaped by a small iris, was focused by a convex lens (L1); then, the light beam passed through a 100-μm pinhole for spatial filtering and was coupled into the double-clad fiber in the multimodal endoscopic probe. A data acquisition (DAQ) card (PCI-5124, National Instrument, Austin, TX, USA) embedded in a computer (PC) with 12-bit resolution and a sampling rate of 200 MS/s was utilized as the data acquisition system. Also, an ultrasound receiver (5073PR, Olympus, Waltham, MA, USA) with an integrated amplifier and a bandwidth of 75 MHz was utilized to receive the photoacoustic signal. The laser exposure was about 8 mJ/cm^2^ at the surface of the tissue, which is lower than the American National Standards Institute (ANSI) laser safety limit (20 mJ/cm^2^).

**Figure 2:**
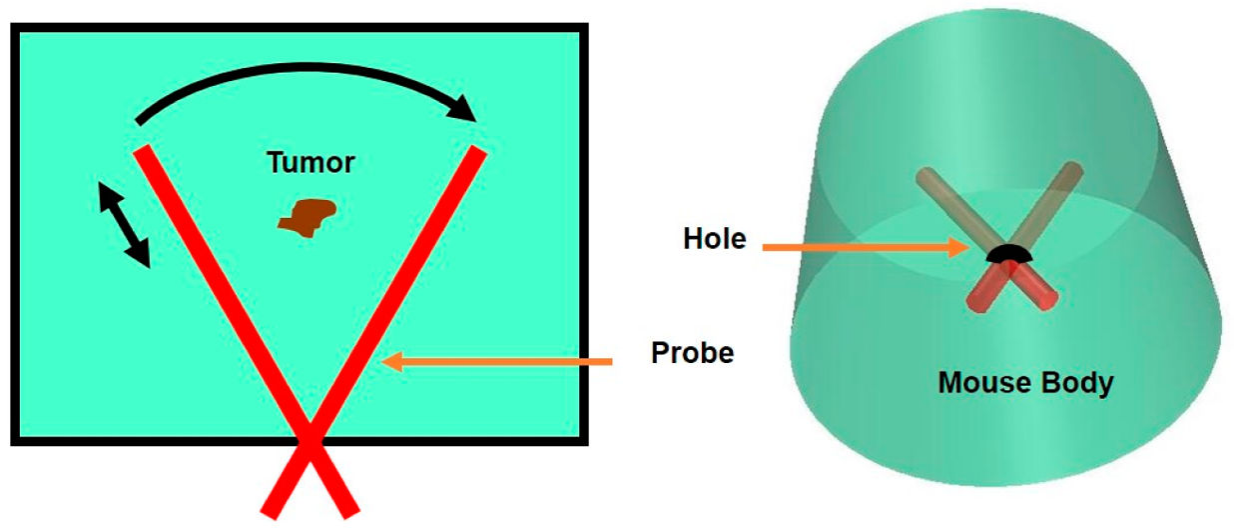
Schematic of the probe scanning mechanism.

To minimize the invasiveness associated with the procedure of plugging the endoscopic probe into the abdomen of a mouse, a novel fan-shaped scanning method was developed. As shown in Figure 2, a hole with a size slightly larger than 1 mm in diameter was drilled through the abdomen of the mouse. A volumetric image of the tissue was obtained by scanning the probe through the tissue in a two-dimensional (2D) fan-shaped plane with the hole location as the center. The 2D fan-shaped scanning (20 mm (radius) *×* 60 degrees) was realized through the combination of a linear translation along radial direction (a step size of 30 μm) and a circular rotation (a step size of 0.3 degree).

### 2.3. Near-Infrared Planar Fluorescence Imaging System

**Figure 3:**
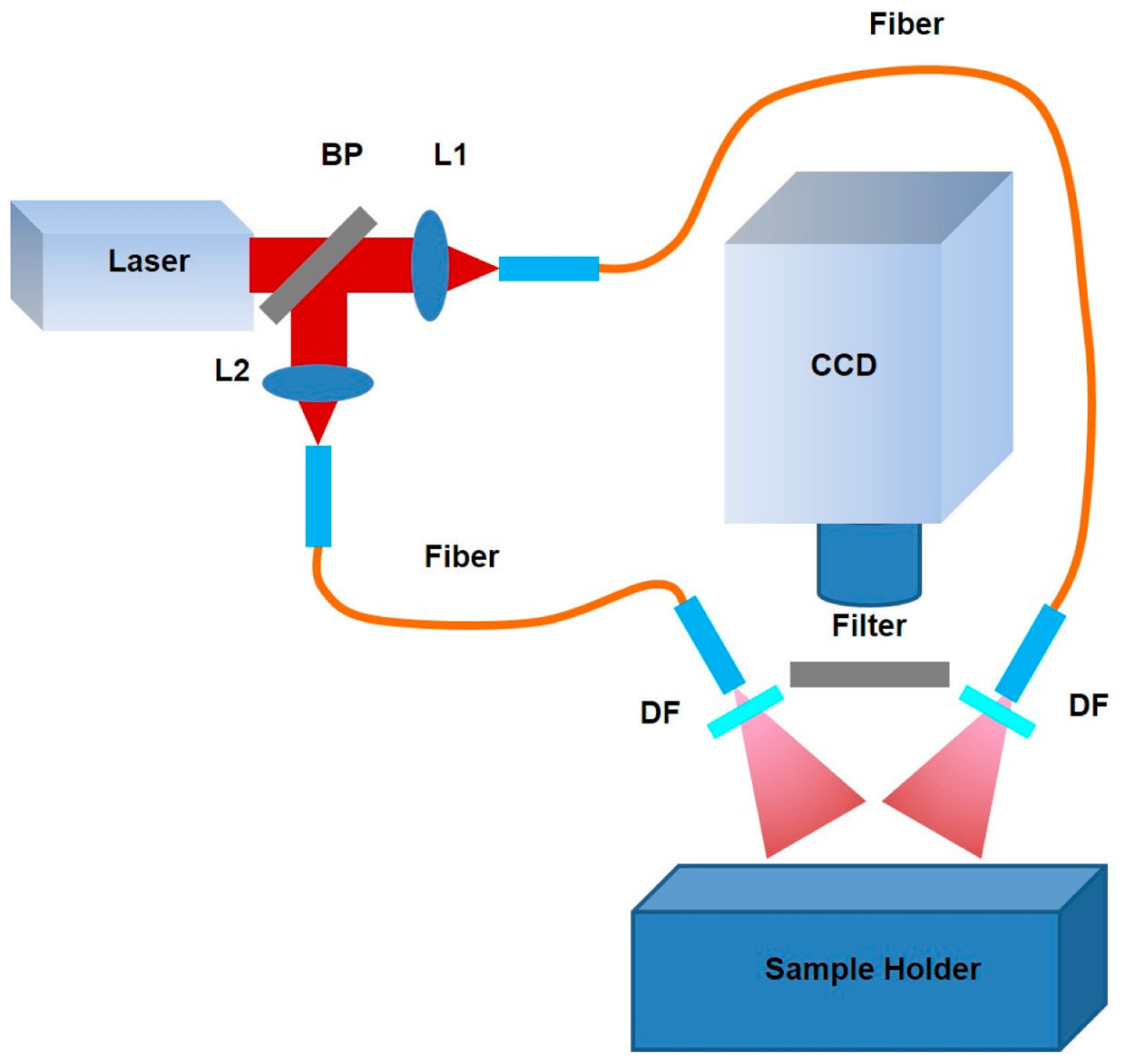
Near-Infrared planar fluorescence imaging system. BP, beam splitter; L1, L2, lens; DF, diffuser.

A conventional near-infrared planar fluorescence imaging system was built to obtain 2D fluorescence images after each endoscopic imaging experiment for further cross validation. As schematically shown in Figure 3, a CW 785 nm laser (M5-785-0080, Thorlabs, Newton, NJ, USA) was used as the light source. The light beam was split into two parts that were respectively coupled into two fiber bundles, and then traveled through light diffusers (DG10-1500, Thorlabs, Newton, NJ, USA) in order to generate homogeneous illumination on the sample mounted on the sample holder. The induced fluorescence signal was collected by a fast charge-coupled device camera (CoolSNAP EZ, Photometrics, Tucson, AZ, USA) with a high performance fluorescent band-pass filter (NT86-381, Edmund Optics, Barrington, NJ, USA) mounted in the front for filtering out non-fluorescence signals. The laser power used for illumination was the same in all experiments.

### 2.4. Image Processing

For endoscopic photoacoustic imaging, the collected photoacoustic signals were first processed through Hilbert transform, and then applied with the time-reversal reconstruction algorithm implemented in Matlab 8.6 to obtain 2D images. These reconstructed 2D images were imported into an image processing platform (Amira 5.4.2) to obtain a 3D volumetric image. The photoacoustic signals from each mouse were calibrated with the laser power used and normalized to the same scale. And all endoscopic photoacoustic images were displayed in the form of maximum amplitude projection (MAP).

For fluorescence imaging, images were collected by a program implemented in National Instruments (NI) Labview, followed by a post-processing tool box in Matlab 8.6. Like photoacoustic imaging, fluorescence signals from each mouse were normalized to the same scale for comparison.

## 3. Results and Discussion

Our previous study [33] showed that the light absorbance of IONPs increases with decreased wavelength; however, considering the fact that tissue penetration depth is greater in the NIR region, light with a wavelength of 730 nm was chosen as the wavelength of the light source for inducing photoacoustic signals in our experiments.

**Figure 4:**
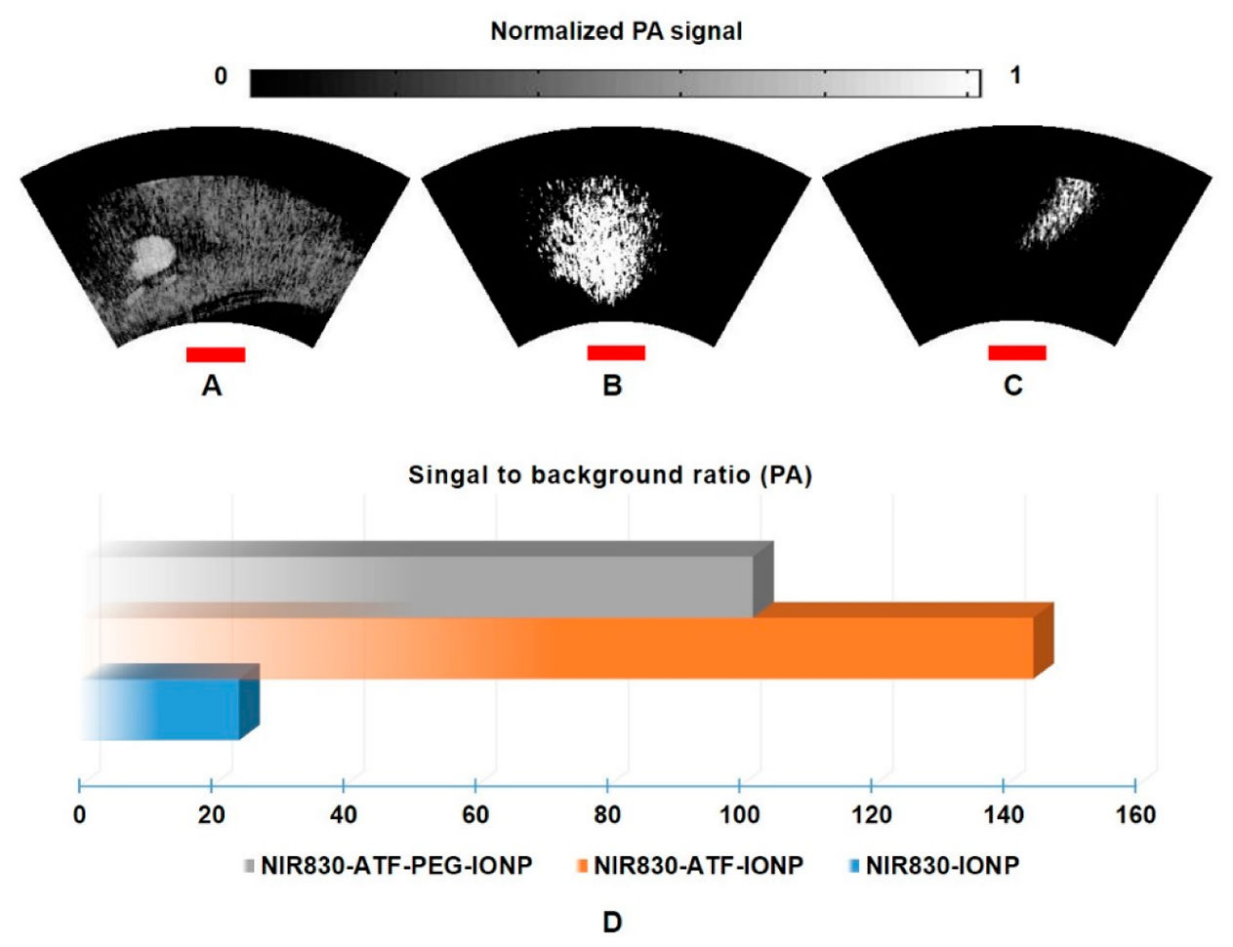
Endoscopic photoacoustic images of pancreatic tumor from the mice of Group 1. Maximum amplitude projection (MAP) images of (**A**) mouse injected with NIR830-IONP; (**B**) mouse injected with NIR830-ATF-IONP; and (**C**) mouse injected with NIR830-ATF-PEG-IONP; (**D**) quantitative plot and comparison of average contrast in region of interest (ROI) (pancreatic tumor). The red scale bars indicate 3 mm in length. NIR, near-infrared; IONP, iron oxide nanoparticle; ATF, amino-terminal fragment; PEG, polyethylene glycol.

Figure 4A–C, respectively, show photoacoustic MAP images from the mice of Group 1 administered with non-targeted nanoparticles NIR830-IONP, targeted NIR830-ATF-IONP, and PEG-coated targeted NIR830-ATF-PEG-IONP. To closely investigate these images, in Figure 4A, we can see that the contrast of tumor to normal tissue is too low to identify the tumor with non-targeted NIR830-IONP. However, for the tumor with targeted NIR830-ATF-IONP or PEG-coated targeted NIR830-ATF-PEG-IONP, greatly enhanced contrast of tumor to normal tissue can be seen in Figure 4B,C, where the tumor area with a clear tumor boundary is pinpointed. In comparing Figure 4B,C, it can be seen that the PEG coating deteriorates the contrast of the tumor to the background, but increases the specificity of tumor detection with a more clearly defined tumor boundary, since PEG was used to stabilize the nanoparticles and to modify surface properties to reduce the non-specific uptake of nanoparticles by macrophages in the reticuloendothelial system. We then selected a region of interest (ROI) within the tumor area, and computed the signal to background ratio (SBR). We plotted the SBR for the mice that had received the injection with three different nanoparticles, as shown in Figure 4D, for quantitative analysis. From these plots, we can see that the mouse with targeted NIR830-ATF-IONP has the highest SBR (~145, 43 dB), while the value for the mouse with non-targeted NIR830-IONP is the lowest (~24, 28 dB). PEG coating reduces the uptake of nanoparticles by cells like macrophages within the tumor, which is indicated by the contrast of the mouse with targeted NIR830-ATF-PEG-IONP (~102, 40 dB).

**Figure 5:**
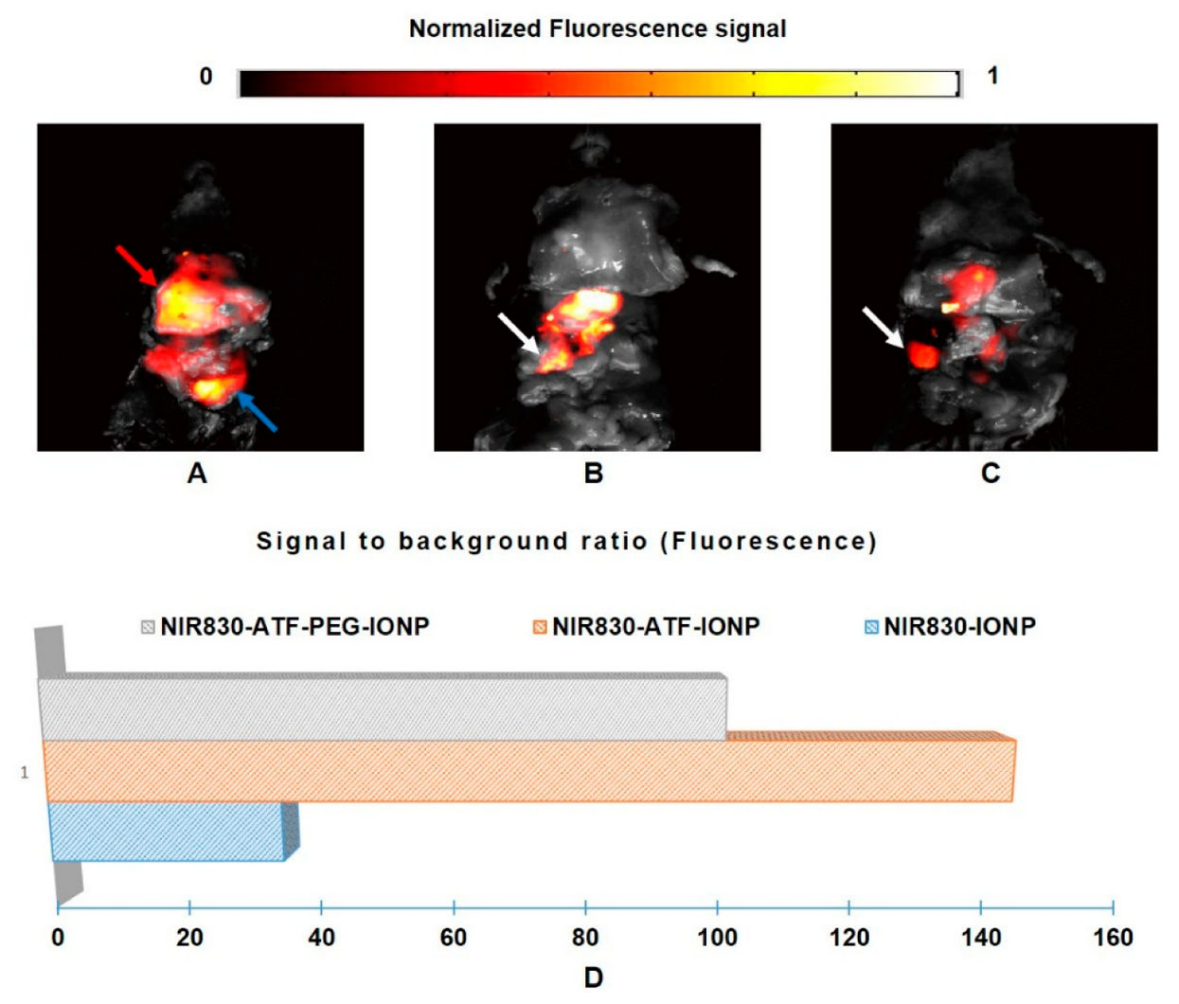
Fluorescence images of pancreatic tumor from the mice of Group 1. Photographs were fused with fluorescence images for (**A**) mouse injected with NIR830-IONP; (**B**) mouse injected with NIR830-ATF-IONP; and (**C**) mouse injected with NIR830-ATF-PEG-IONP; (**D**) quantitative plot and comparison of average contrast in region of interest (ROI) (pancreatic tumor).

After the endoscopic photoacoustic imaging, we then conducted experiments using the NIR planar fluorescence imaging system. Figure 5A–C show the fluorescence images from the mice of Group 1 injected with NIR830-IONP, NIR830-ATF-IONP, and NIR830-ATF-PEG-IONP, respectively. The highest contrast in the image for the mouse with non-targeted NIR830-IONP (Figure 5A) comes from both the tumor (indicated by red arrow) and normal tissue (indicated by blue arrow). Thus, the specificity is too low to identify the tumor. On the contrary, for the mouse with the targeted agent of NIR830-ATF-IONP (Figure 5B) or NIR830-ATF-PEG-IONP (Figure 5C), greatly enhanced image contrast was observed, allowing the tumor and the tumor boundary to be identified clearly. Taking a close look at the difference between the images for NIR830-ATF-IONP and NIR830-ATF-PEG-IONP, we note that the PEG coating reduces tissue uptake of nanoparticles, especially in organs without tumors, as indicated by the white arrows in Figure 5B,C. We also plotted the SBR of the ROI from these three mice, as shown in Figure 5D, where we see SBR values similar to those of the photoacoustic images (Figure 4D): The mouse with NIR830-IONP has a SBR of ~35 (31 dB), while the mice with NIR830-ATF-IONP or NIR830-ATF-PEG-IONP, respectively, have an SBR of ~144 (43 dB) and ~101 (40 dB).

**Figure 6:**
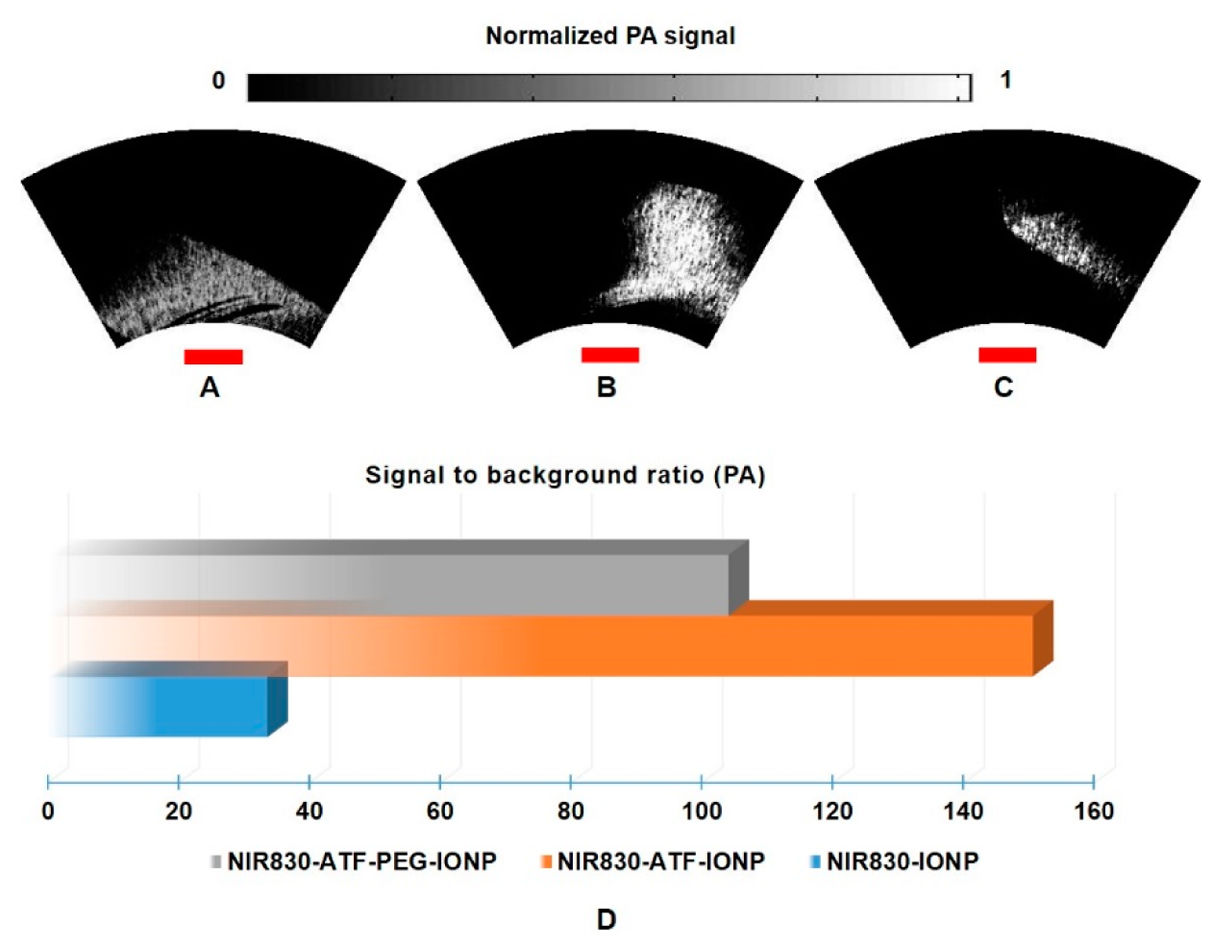
Endoscopic photoacoustic images of pancreatic tumor from the mice of Group 2. Maximum amplitude projection (MAP) images of (**A**) mouse injected with NIR830-IONP; (**B**) mouse injected with NIR830-ATF-IONP; and (**C**) mouse injected with NIR830-ATF-PEG-IONP; (**D**) quantitative plot and comparison of average contrast in region of interest (ROI) (pancreatic tumor). The red scale bars indicate 3 mm in length.

**Figure 7:**
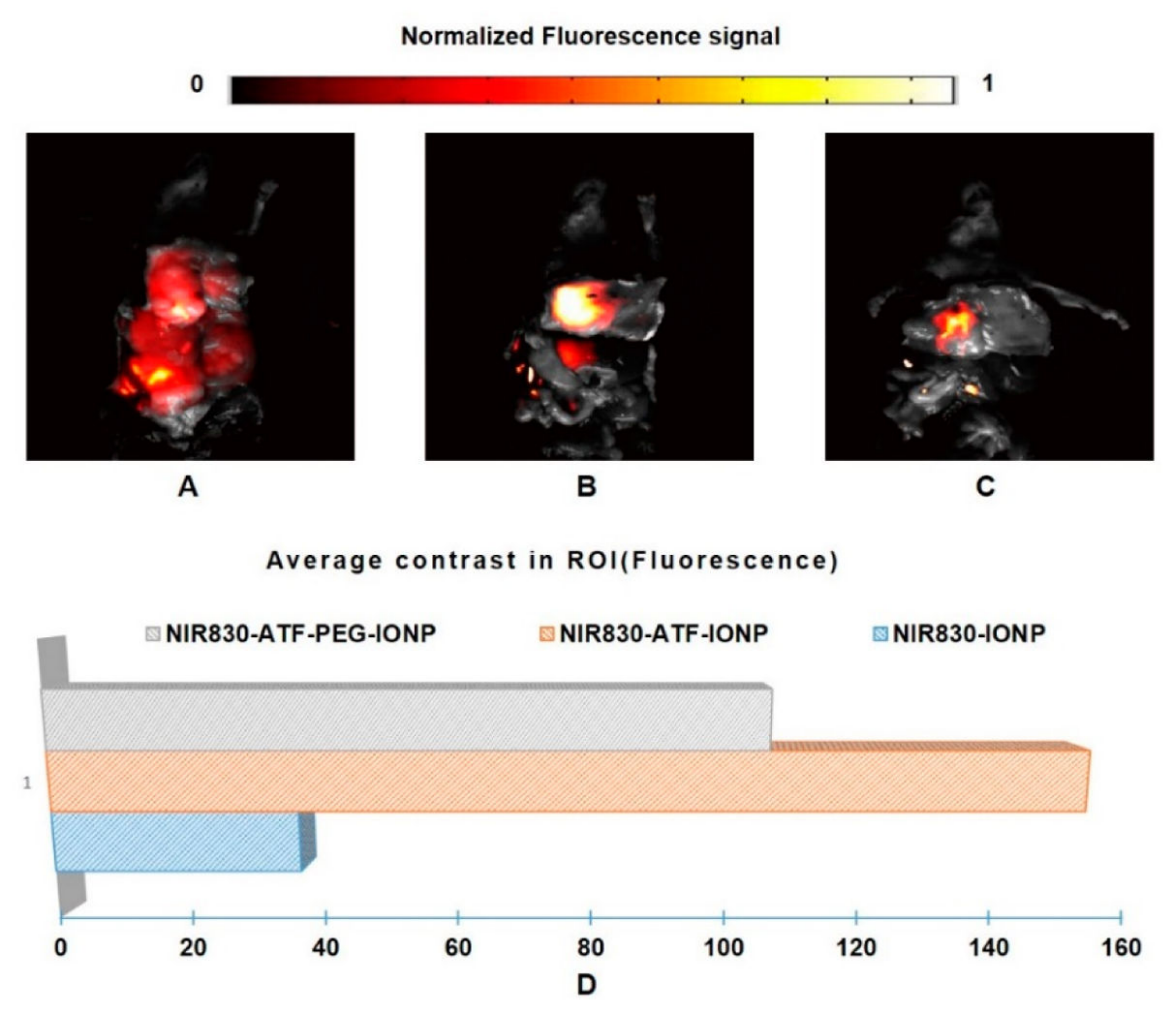
Fluorescence images of pancreatic tumor from the mice of Group 2. Photographs were fused with fluorescence images from (**A**) mouse injected with NIR830-IONP; (**B**) mouse injected with NIR830-ATF-IONP; and (**C**) mouse injected with NIR830-ATF-PEG-IONP; (**D**) quantitative plot and comparison of average contrast in region of interest (ROI) (pancreatic tumor).

The endoscopic photoacoustic images and quantitative plots for the second group of mice are shown in Figure 6, while the corresponding fluorescence images and plots are shown in Figure 7. These images show image quality that is comparable to that seen in the first group of mice.

## 4. Conclusions

This work represents the first report on multimodal photoacoustic-fluorescence molecular endoscopic imaging of pancreatic cancer using a miniature probe and targeted multifunctional magnetic iron oxide nanoparticles in a mouse pancreatic cancer model. With the novel fan-shaped scanning mechanism, the endoscopic probe can directly reach the surface of the pancreas through the abdomen with minimal invasiveness. The photoacoustic and fluorescence signals of the tumor region were significantly improved with targeted multifunctional nanoparticles. This study indicates the potential of targeted multimodal photoacoustic-fluorescence endoscopic molecular imaging for the early detection of pancreatic cancer. Before the approach can be used for in vivo studies, questions concerning barriers from blood or vessels surrounding the tumor area should be tackled, which we plan to study in the future.
